# Associations Between Sleep Duration, Wake-Up Time, Bedtime, and Abdominal Obesity: Results From 9559 Chinese Children Aged 7–18 Years

**DOI:** 10.3389/fendo.2021.735952

**Published:** 2021-10-14

**Authors:** Meijuan Liu, Bingyan Cao, Qipeng Luo, Qiao Wang, Min Liu, Xuejun Liang, Di Wu, Wenjing Li, Chang Su, Jiajia Chen, Chunxiu Gong

**Affiliations:** ^1^ Department of Endocrinology, Genetics and Metabolism, Beijing Children’s Hospital, Capital Medical University, National Center for Children’s Health, Beijing, China; ^2^ Department of Pain Medicine, Peking University Third Hospital, Beijing, China

**Keywords:** children, abdominal obesity, sleep duration, wake-up time, bedtime, weekend

## Abstract

**Objective:**

To investigate the associations of sleep duration, wake-up time, bedtime, and childhood abdominal obesity, and to test whether there is a weekday/weekend difference and the potential modifying role of sex.

**Methods:**

This cross-sectional study was based on the Students’ Constitution and Health Survey and included 9559 students (4840 boys and 4719 girls) aged 7–18 years (7227 aged 7–12 years, 2332 aged 13–18 years). They were divided into two groups (control group and group with abdominal obesity). The physical measurements included children and youth body height, body weight, and waist circumference (WC). A parent-report questionnaire was used to collect the information about parent characteristics as well as lifestyle and sleep patterns (sleep duration, bedtime, and wake-up time of weekdays and weekends) of children and youth.

**Results:**

The prevalence of abdominal obesity was 30.57% and the percentages of sleep duration <9 hours/day, wake-up time before 07:00 am on weekdays and weekends, bedtime after 10:00 pm on weekends were significantly higher in the group with abdominal obesity. After adjusting for confounders, sleep duration <9 hours/day on weekends was inversely related to abdominal obesity in the overall subjects, regardless of their sex and age, while bedtime after 10:00 pm on weekends was inversely related to abdominal obesity only in the overall subjects, boys, and children aged 7–12 years. Logistic regression models in all subjects showed that shorter hours of weekends sleep duration were associated with greater risks of abdominal obesity, even after adjusting for all confounders, including wake-up time and bedtime. The adjusted odds ratios and 95% confidence intervals of abdominal obesity (with ≥10 hours/day as the reference group) for children with 9–10 hours/day, 8–9 hours/day, and <8 hours/day of weekend sleep duration were 1.23 (1.04–1.46), 1.59 (1.32–1.91) and 1.83 (1.42–2.36), respectively. Specifically, after stratification by sex and age, this phenomenon was only observed in boys and children aged 7–12 years.

**Conclusions:**

Sleep duration and bedtime on weekends were independently associated with the risk of childhood abdominal obesity, particularly in boys and children aged 7–12 years.

## Introduction

Childhood obesity has become a serious public health issue, especially in developing countries, like China (1, 2). According to data from the Report on Childhood Obesity in China in 2017, the number of children and youth with overweight or obesity increased from 6.15 million in 1985 to 34.96 million in 2014 (3, 4). Childhood obesity has serious short-and long-term consequences for physical and psychosocial health, including heart disease, hypertension, stroke, type 2 diabetes, metabolic syndrome, low self-esteem, negative self-evaluation, decreased self-image, social isolation, social discrimination, etc. (5). In addition to the influences on the health of individuals, childhood obesity also puts a major economic burden on governmental health organizations (5). Importantly, abdominal obesity, also known as central obesity is more harmful than general obesity. The risk of hypertension, dyslipidemia, and metabolic syndrome was significantly higher in patients with abdominal obesity than in those with general obesity (6). Both unmodifiable factors (e.g., genetic, endocrine disorders) and modifiable factors (e.g., parental determinants, individual lifestyle, socioeconomic status) influence the risk of childhood obesity (7). Therefore, it is imperative to identify modifiable risk factors to reduce the burden of childhood obesity and its adverse consequences.

Sleep is one of the modifiable behaviors and plays an important role in childhood obesity. Short sleep duration is very common in modern society, with approximately one–third to two–thirds of adolescents complain about insufficient sleep (8–10). Although emerging evidences from both cross-sectional and longitudinal studies have indicated the close relationship between short sleep duration and obesity (11–13), including in Chinese children (14–17), studies focusing on the relationship between sleep duration and abdominal obesity are still limited. Recently, several studies found that sleeping longer on weekends may help ameliorate some of the effects of insufficient sleep during the weekdays, which suggested that weekday-weekend variability in sleep duration may play a role in overweight and obesity (18, 19). Moreover, sex differences in sleep duration have been reported among children and adolescents (20–22). Therefore, examining the relationship between sleep duration and abdominal obesity and exploring the potential weekday/weekend and sex differences can assist with developing more targeted interventions against abdominal obesity. However, to our knowledge, few studies have focused on weekday/weekend and sex differences in examining the association between childhood abdominal obesity and sleep duration.

In recent years, sleep timing, including wake-up time and bedtime, is another important modifiable behavior, which is closely related to childhood obesity. Although there were many studies regarding the associations between wake-up time, bedtime, and childhood obesity, the results were inconsistent (11, 23, 24). Additionally, few studies on the associations of wake-up time, bedtime with abdominal obesity among Chinese children were available.

Therefore, this study aimed to investigate the associations between sleep duration, wake-up time, bedtime, separately on weekdays and weekends, and childhood abdominal obesity. Moreover, since age-specific and sex-specific associations have been reported in the literature between sleep duration and childhood obesity (13, 23), these associations regarding sex and age differences were also explored.

## Materials and Methods

### Study Population

This was a cross-sectional, school-based study, which used data from the survey on Students’ Constitution and Health that was carried out in Beijing, the capital of China. In brief, a physical examination and a questionnaire survey were conducted in this study.

A total of 9559 students (4840 boys and 4719 girls) aged 7–18 years (3803 boys and 3424 girls aged 7–12 years, 1037 boys and 1295 girls aged 13–18 years) from nine primary schools and five secondary schools were recruited in this study. They were divided into two groups, the control group (n=6637) and the group with abdominal obesity (n=2922). This study received approval from the ethics committee of Beijing Children’s Hospital, Capital Medical University. All subjects were informed about the aims and procedures of the study and written informed consents were received from all children’s parents before they participating in the study.

### Anthropometric Measurements and Definitions

Children and youth height, body weight, and waist circumference (WC) were measured by the well-trained research staff. Height was measured to the nearest 0.1 cm with subjects wearing no shoes, and weight was measured to the nearest 0.1 kg with subjects wearing lightweight clothing. WC was measured to the nearest 0.1 cm at the midpoint between the inferior costal margin and the superior border of the iliac crest on the midaxillary line. Two measurements (measurement error ≤ 1 mm) were obtained and the average was used for the analysis. Body mass index (BMI) was calculated as weight (kg) divided by height squared (m^2^). Abdominal obesity was defined as WC > 90–th percentile (25), which according to age- and sex-specific WC cut-off points recommended by Ji et al. (26). Parental height and weight were acquired from the questionnaires. Parental obesity was defined as BMI ≥28 kg/m^2^, which was recommended by the Working Group for Obesity in China (WGOC) (27).

### Questionnaire

The questionnaire information regarding each subject was collected by the trained investigators through a face-to-face interview with at least one parent. The questionnaire included questions as follows: age, sex, parental height, parental weight, parental education level (primary school or junior high school or senior high school or college or graduate or above), gestational diabetes mellitus (yes or no), infant breastfeeding (yes or no), birth weight, having one or more siblings (yes or no), household income (<20 ten thousand yuan/year or ≥20 ten thousand yuan/year), watching TV (<2 hours/day or ≥2 hours/day), frequency of exercise (≤3 times/week or >3 times/week), exercise time (<120 minutes/week or ≥120 minutes/week), wake-up time (before 06:00 am or 06:00-07:00 am or 07:00-08:00 am or 08:00-09:00 am or after 09:00 am), bedtime (before 08:00 pm or 08:00-09:00 pm or 09:00-10:00 pm or 10:00-11:00 pm or 11:00-12:00 pm or after midnight), number of nocturnal awakenings (≤2 times/month or >2 times/month), and frequency of snoring (≤2 times/month or >2 times/month). In this study, parental education level was classified into two categories: below college (primary/junior/senior high school), and college or more (college or graduate or above). Wake-up time and bedtime were also classified into two categories: before 07:00 am/after 07:00 am and before 10:00 pm/after 10:00 pm, respectively. Sleep duration was calculated by using parent’s reports of wake-up time and bedtime and subsequently classified into two categories: <9 hours/day and ≥9 hours/day. The sleep duration, bedtime, and wake-up time were assessed separately for weekdays and weekends.

### Statistical Analysis

All values were expressed as mean ± standard deviation (SD) or number (percentages), as appropriate. The comparisons of continuous and categorical variables were analyzed by the independent t-test and chi-square tests, respectively. Then, the associations among the independent variable (abdominal obesity) and dependent variables (age, sex/gender, parental BMI, parental education level, gestational diabetes mellitus, infant breastfeeding, birth weight, siblings, household income, watching TV, frequency of exercise, exercise time, sleep duration, wake-up time, bedtime, number of nocturnal awakenings, and frequency of snoring) were examined using bivariate logistic regression models. The odds ratios (ORs) and the 95% confidence intervals (CIs) were obtained. Analyses were repeated stratified by age groups (7–12 and 13–18 years) and sex/gender, respectively. Finally, sleep duration was used as the ordered categorical variable (<8 hours/day, 8–9 hours/day, 9–10 hours/day, >10 hours/day) and its association with abdominal obesity was explored with univariate and multivariate logistic regression analyses. Model 1 was the basic model, adjusted for age and sex, while model 2 further adjusted for parental obesity, parental education level, gestational diabetes mellitus, infant breastfeeding, birth weight, siblings, and household income. The final model, model 3 further adjusted for watching TV, frequency of exercise, exercise time, sleep duration (weekdays), wake-up time (weekdays), bedtime (weekdays), wake-up time (weekends), bedtime (weekends), number of nocturnal awakenings, and frequency of snoring based on model 2. Subgroup analyses by age groups (7–12 and 13–18 years) and sex were also performed. All analyses were conducted using the SPSS version 20.0 for Windows (SPSS Inc, Chicago, IL, USA) and *p*-value <0.05 was statistically significant.

## Results

### Baseline Characteristics of Study Participants

The baseline characteristics of all children were presented in **Table 1**. A total of 9559 children (50.6% boys, aged 10.7 ± 2.81 years) were included in the present study. The prevalence of abdominal obesity was 30.57%. Children with abdominal obesity were significantly older, with higher percentages of boys, parents with obesity, low parental education (below college), gestational diabetes mellitus, birth weight ≥4 kg, low household income (<20 ten thousand yuan), less frequency (≤3 times/week) and less time of exercise (<120 minutes/week), snoring (>2 times/month), but lower percentages of infant breastfeeding and having one or more siblings, when compared with controls (*p* all <0.05). Additionally, a larger proportion of children with abdominal obesity had sleep duration<9 hours/day as compared to controls, both on weekdays and weekends (*p* all <0.05). Moreover, the proportions of children who used to sleep late on weekends (after 10:00 pm), but get up early on weekdays and weekends (before 07:00 am) were larger in children with abdominal obesity (*p* all <0.05).

**Table 1:** Baseline characteristics of study participants.

	Control (n = 6637)	Abdominal obesity (n = 2922)	All (n = 9559)	p^*^
**Age (years)**	10.6 ± 2.87	10.9 ± 2.67	10.7 ± 2.81	**<0.01**
**Sex**				
**Boy**	3133 (47.2%)	1707 (58.4%)	4840 (50.6%)	**<0.01**
**Girl**	3504 (52.8%)	1215 (41.6%)	4719 (49.4%)	
**Father obesity**				
**Yes**	1230 (18.5%)	945 (32.3%)	2175 (22.8%)	**<0.01**
**No**	5407 (81.5%)	1977 (67.7%)	7384 (77.2%)	
**Father’s education**				
**Below college**	2896 (43.6%)	1382 (47.3%)	4278 (44.8%)	**<0.01**
**College or more**	3741 (56.4%)	1540 (52.7%)	5281 (55.2%)	
**Mother obesity**				
**Yes**	411 (6.2%)	426 (14.6%)	837 (8.8%)	**<0.01**
**No**	6226 (93.8%)	2496 (85.4%)	8722 (91.2%)	
**Mother’s education**				
**Below college**	2782 (41.9%)	1301 (44.5%)	4083 (42.7%)	**<0.05**
**College or more**	3855 (58.1%)	2289 (56.5%)	5476 (57.3%)	
**Gestational diabetes mellitus**				
**Yes**	403 (6.1%)	237 (8.1%)	640 (6.7%)	**<0.01**
**No**	6234 (93.9%)	2685 (91.9%)	8919 (93.3%)	
**Infant breastfeeding**				
**Yes**	4905 (73.9%)	2089 (71.5%)	6994 (73.2%)	**<0.05**
**No**	1732 (26.1%)	833 (28.5%)	2565 (26.8%)	
**Birth weight ≥4 kg**				
**Yes**	825 (12.4%)	495 (16.9%)	1320 (13.8%)	**<0.01**
**No**	5812 (87.6%)	2427 (83.1%)	8239 (86.2%)	
**Household income (year)**				
**<20 ten thousand yuan**	5020 (75.6%)	2296 (78.6%)	7316 (76.5%)	**<0.01**
**≥20 ten thousand yuan**	1617 (24.4%)	626 (21.4%)	2243 (23.5%)	
**Having 1 or more siblings**				
**Yes**	2262 (34.1%)	838 (28.7%)	3100 (32.4%)	**<0.01**
**No**	4375 (65.9%)	2084 (71.3%)	6459 (67.6%)	
**Watching TV**				
**<2 hours/day**	5902 (88.9%)	2516 (86.1%)	8418 (88.1%)	0.11
**≥2 hours/day**	735 (11.1%)	406 (13.9%)	1141 (11.9%)	
**Frequency of exercise**				
**≤3 times/week**	3717 (56.0%)	1881 (64.4%)	5598 (58.6%)	**<0.01**
**>3 times/week**	2920 (44.0%)	1041 (35.6%)	3961 (41.4%)	
**Exercise time**				
**<120 minutes/week**	3685 (55.5%)	1839 (62.9%)	5524 (57.8%)	**<0.01**
**≥120 minutes/week**	2952 (44.5%)	1083 (37.1%)	4035 (42.2%)	
**Sleep duration (weekdays)**				
**<9 hours/day**	5932 (89.4%)	2658 (91.0%)	8590 (89.9%)	**<0.05**
**≥9 hours/day**	705 (10.6%)	264 (9.0%)	969 (10.1%)	
**Wake-up time (weekdays)**				
**Before 07:00 am**	6445 (97.1%)	2860 (97.9%)	9305 (97.3%)	**<0.05**
**After 07:00 am**	192 (2.9%)	62 (2.1%)	254 (2.7%)	
**Bedtime (weekdays)**				
**Before 10:00 pm**	4245 (64.0%)	1811 (62.0%)	6056 (63.4%)	0.07
**After 10:00 pm**	2392 (36.0%)	1111 (38.0%)	3503 (36.6%)	
**Sleep duration (weekends)**				
**<9 hours/day**	3211 (48.4%)	1704 (58.3%)	4915 (51.4%)	**<0.01**
**≥9 hours/day**	3426 (51.6%)	1218 (41.7%)	4644 (48.6%)	
**Wake-up time (weekends)**				
**Before 07:00 am**	1493 (22.5%)	793 (27.1%)	2286 (23.9%)	**<0.01**
**After 07:00 am**	5144 (77.5%)	2129 (72.9%)	7273 (76.1%)	
**Bedtime (weekends)**				
**Before 10:00 pm**	2865 (43.2%)	1108 (37.9%)	3973 (41.6%)	**<0.01**
**After 10:00 pm**	3772 (56.8%)	1814 (62.1%)	5586 (58.4%)	
**Number of nocturnal awakenings**				
**≤2 times/month**	5809 (87.5%)	2565 (87.8%)	8374 (87.6%)	0.75
**>2 times/month**	828 (12.5%)	357 (12.2%)	1185 (12.4%)	
**Frequency of snoring**				
**≤2 times/month**	5892 (88.8%)	2180 (74.6%)	8072 (84.4%)	**<0.01**
**>2 times/month**	745 (11.2%)	742 (25.4%)	1487 (15.6%)	

below college, primary/junior/senior high school; college or more, college or graduate or above; TV, television. The data were presented as the mean ± SD for continuous variables or as the number (%) for categorical variables. ^*^The p-values were obtained from the independent t-test for continuous variables and chi-square tests for categorical variables. Statistically significant (p < 0.05) were marked in bold.

### Bivariate Logistic Regression Analysis of Sleep Duration, Wake-Up Time, Bedtime, and Abdominal Obesity

**Figure 1:**
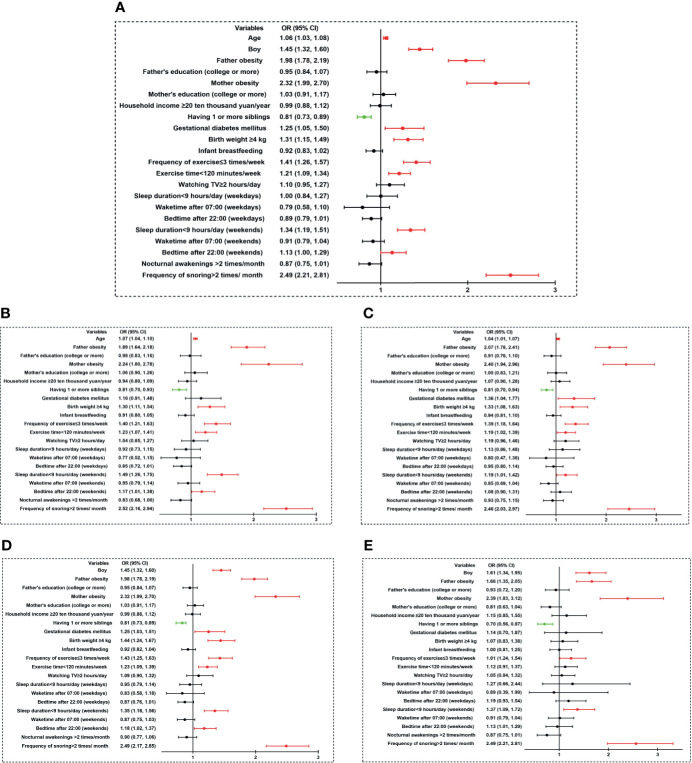
Bivariate logistic regression analysis of sleep duration, sleep timing, and abdominal obesity. **(A)** all children; **(B)** boys; **(C)** girls; **(D)** children aged 7–12 years; **(E)** children aged 13–18 years). Variables that were significantly positively associated with abdominal obesity risk were shown in red color (*p* < 0.05). Variables that were significantly negatively associated with abdominal obesity risk were shown in green color (*p* < 0.05). OR, odds ratio; CI, confidence interval; TV, television.

The bivariate logistic regression analysis of sleep duration, wake-up time, bedtime, and abdominal obesity was shown in **Figure 1**. In all subjects, age, gender, father obesity, mother obesity, gestational diabetes mellitus, birth weight ≥4 kg, frequency of exercise ≤ 3 times/week, exercise time<120 minutes/week, sleep duration <9 hours/day on weekends, bedtime after 10:00 pm on weekends, and frequency of snoring>2 times/month were independently positively associated with abdominal obesity, whilst having one or more siblings was negatively associated with abdominal obesity (*p* all <0.05) (**Figure 1A**).

As shown in **Figures 1B, C**, when data was split by sex and analyzed again: age, father obesity, mother obesity, birth weight ≥4 kg, frequency of exercise ≤ 3 times/week, exercise time<120 minutes/week, sleep duration <9 hours/day on weekends, frequency of snoring>2 times/month were also positively associated with abdominal obesity, while having one or more siblings was negatively associated with abdominal obesity in both genders (*p* all <0.05). Bedtime after 10:00 pm on weekends was independently positively associated with abdominal obesity in boys (OR=1.17, 95% CI 1.01–1.38), but not in girls (OR=1.08, 95% CI 0.90–1.31). Gestational diabetes mellitus was independently positively associated with abdominal obesity in girls (OR=1.36, 95% CI 1.04–1.77), but not in boys (OR=1.16, 95% CI 0.91–1.48).

When the subgroup analysis was conducted between children aged 7–12 years and children aged 13–18 years, as shown in **Figures 1D, E**, abdominal obesity was positively associated with sex, father obesity, mother obesity, frequency of exercise ≤ 3 times/week, sleep duration <9 hours/day on weekends, frequency of snoring >2 times/month, but negatively associated with having one or more siblings in both children aged 7–12 years and children aged 13–18 years (*p* all <0.05). It was observed that gestational diabetes mellitus (OR=1.25, 95% CI 1.03–1.51), birth weight ≥4 kg (OR=1.44, 95% CI 1.24–1.67), exercise time<120 minutes/week (OR=1.23, 95% CI 1.09–1.39), and bedtime after 22:00 on weekends (OR=1.18, 95% CI 1.02–1.37) were only independently positively associated with abdominal obesity in children aged 7–12 years.

### Associations Between Weekend Sleep Duration and Abdominal Obesity

As mentioned above, weekend sleep duration was independently associated with childhood abdominal obesity, whether in the overall analysis or stratified by age or sex. We further explored its relationship with abdominal obesity when sleep duration was used as the ordered categorical variable (<8 hours/day, 8–9 hours/day, 9–10 hours/day, >10 hours/day). As shown in **Table 2**, we found that shorter weekend sleep duration was associated with an increased risk of abdominal obesity. In the univariate model, the odds of being abdominal obesity increased by 24.3%, 66.8%, 106.5% in children with weekend sleep duration 9–10 hours/day, 8–9 hours/day, <8 hours/day, respectively, in comparison with children with weekend sleep duration>10 hours/day (*p* all <0.05). This association remained significant even after various adjustments were made (**Supplementary Table 1**). Even after adjusting all variables in Model 3, including wake-up time and bedtime, the decreasing weekend sleep duration was still associated with increased risk of abdominal obesity, as children who had short weekend sleep duration (9–10 hours/day, 8–9 hours/day, <8 hours/day) were at greater risk of developing abdominal obesity (OR=1.23, 95% CI 1.04–1.46; OR=1.59, 95% CI 1.32–1.91; OR=1.83, 95% CI 1.42–2.36, respectively).

**Table 2:** Unconditional logistic regression analysis of associations between weekend sleep duration and abdominal obesity.

	weekend sleep duration, hours			
Measurement	<8 hours/day OR (95% CI)	8–9 hours/day OR (95% CI)	9–10 hours/day OR (95% CI)	>10 hours/day OR (95% CI)
Overall				
Univariate	2.07 (1.73–2.47)	1.67 (1.43–1.95)	1.24 (1.03–1.46)	1.00 (reference)
Model 3	1.83 (1.42–2.36)	1.59 (1.32–1.91)	1.23 (1.04–1.46)	1.00 (reference)
Boys				
Univariate	2.21 (1.72–2.84)	1.76 (1.41–2.20)	1.22 (0.97–1.52)	1.00 (reference)
Model 3	2.24 (1.57–3.20)	1.84 (1.41–2.39)	1.28 (1.01–1.63)	1.00 (reference)
Girls				
Univariate	1.77 (1.36–2.29)	1.45 (1.16–1.81)	1.24 (0.99–1.54)	1.00 (reference)
Model 3	1.51 (1.05–2.17)	1.38 (1.06–1.79)	1.20 (0.95–1.52)	1.00 (reference)
7–12 years old				
Univariate	2.53 (2.03–3.16)	1.71 (1.43–2.04)	1.27 (1.06–1.52)	1.00 (reference)
Model 3	2.11 (1.54–2.91)	1.60 (1.29–1.99)	1.25 (1.03–1.51)	1.00 (reference)
13–18 years old				
Univariate	1.53 (1.10–2.13)	1.54 (1.12–2.12)	1.17 (0.84–1.62)	1.00 (reference)
Model 3	1.54 (1.00–2.41)	1.59 (1.10–2.29)	1.23 (0.86–1.76)	1.00 (reference)

Multivariate ORs and 95% CIs from unconditional logistic regression models were used in the analysis.

Model 3: full model, adjusted for age, gender, parental obesity, parental education, gestational diabetes mellitus, infant breastfeeding, birth weight, siblings, household income, watching TV, frequency of exercise, exercise time, sleep duration (weekdays), wake-up time (weekdays), bedtime (weekdays), wake-up time (weekends), bedtime (weekends), number of nocturnal awakenings, frequency of snoring.

Additionally, subgroup analysis was performed according to sex. We found that after adjusting all variables in Model 3, the increased risk of abdominal obesity with decreasing weekend sleep duration was revealed in boys (OR=1.28, 95% CI 1.01–1.63; OR=1.84, 95% CI 1.41–2.39; OR=2.24, 95% CI 1.57–3.20 for 9–10 hours/day, 8–9 hours/day, <8 hours/day, respectively), but was much weaker in girls. Specifically, children with weekend sleep duration 8–9 hours/day and <8 hours/day were associated with higher risks of abdominal obesity in both genders, when compared to the reference group (>10 hours/day), even after adjusting all variables in Model 3 (OR=1.84, 95% CI 1.41–2.39; OR=2.24, 95% CI 1.57–3.20 for boys, OR=1.38, 95% CI 1.06–1.79; OR=1.51, 95% CI 1.05–2.17 for girls, respectively) (**Table 2**).

Moreover, we also performed subgroup analysis according to age (7–12 years, 13–18 years). After including all variables in Model 3, the phenomenon that the odds of abdominal obesity steadily increased with the decreasing weekend sleep duration was observed in children aged 7–12 years (OR=1.25, 95% CI 1.03–1.51; OR=1.60, 95% CI 1.29–1.99; OR=2.11, 95% CI 1.54–2.91 for 9–10 hours/day, 8–9 hours/day, <8 hours/day, respectively), but was much weaker in children aged 13–18 years (**Table 2**).

## Discussion

In this cross-sectional study with a large sample of Chinese children aged 7–18 years, we found that short sleep duration (<9 hours/day) and late bedtime (after 10:00 pm) were associated with an increased risk of childhood abdominal obesity, although this was only significant at weekends, not weekdays. This study also found that the decreasing weekend sleep duration was associated with an increased risk of abdominal obesity, when sleep duration was analyzed as the ordered categorical variable (<8 hours/day, 8–9 hours/day, 9–10 hours/day, >10 hours/day). Noteworthy, this phenomenon was more prominent for boys and children aged 7–12 years than for girls and children aged 13–18 years, respectively. To our knowledge, this is one of the few studies that investigated not only the sleep duration but also wake-up time and bedtime on weekdays and weekends in association with childhood abdominal obesity, with a special emphasis on age and sex differences.

A series of studies have provided strong evidence of an association between sleep duration and obesity in children (12–16, 23). Our study found that short weekend sleep duration (OR for less than 9 hours) was associated with childhood abdominal obesity, even after adjusting for all confounders, including sleep timing wake-up time and bedtime. This association remained after the subgroup analyses of sex and age were conducted. Consistent with our findings, a cross-sectional study conducted by Yu et al. among 500 Chinese adolescent twins reported that the association between short sleep duration and obesity is most likely mediated by total and central adiposity (28). Short sleep duration has been associated with abdominal obesity in 1008 Chinese children aged 6–17 years (20), as well as in 6048 Korea participants aged 10–18 years (12). Moreover, a recent meta-analysis of 22 studies with a total of 56259 participants demonstrated a negative association between sleep duration and WC, indicating the close association between sleep duration and abdominal obesity (29). However, it should be noted that the previous studies did not distinguish the association of sleep duration on abdominal obesity between weekdays and weekends.

Since children spend most of their time at school or the after-school academic institutes and have a huge amount of homework on weekdays, the sleep duration and timing on weekdays and weekends are quite different (30). Our study found that weekend sleep duration, but not weekday sleep duration, was associated with childhood abdominal obesity. In support of our results, a recent epidemiological study in 1728 children in Greece found that the association between sleep duration and overweight/obesity was stronger on weekends than on weekdays (11). Im et al. (31) and Kim et al. (32) also revealed that sleep longer on weekends may have protective effects in preventing childhood obesity. Although the detailed mechanisms are unclear yet, one possible explanation is that weekend sleep duration time might serve as a buffer that attenuates the detrimental impact of weekday sleep insufficiency on obesity, and facilitates an efficient recovery from energy dysregulation (32, 33). Further studies are needed to specifically address the underlying mechanism.

Notably, when weekend sleep duration time was analyzed as the ordered categorical variable (<8 hours/day, 8–9 hours/day, 9–10 hours/day, >10 hours/day), a linear dose-response relationship was found, indicating that the shorter weekend sleep duration, the higher risk of having abdominal obesity. The linear relationship between sleep duration and childhood overweight/obesity was also observed in several previous studies. Lytle et al. studied in 723 American adolescents found that with each additional hour of sleep per day lost, the risk of being obese increased by 80% (21). Similarly, a cross-sectional study involving 529 students from the USA observed a “dose-response” relationship between sleep duration and overweight, with increasing adjusted odds ratios of overweight with decreasing sleep duration (34). However, there were also many studies revealing a U-shape association between sleep duration and overweight/obesity (17, 35–37). For example, in analysis among 66817 Chinese adolescents in grades 7–12, Wu et al. found that when 7.0–9.0 hours/day of sleep considered as the reference group, the adjusted ORs of overweight/obesity were higher in groups of short sleep (< 5.0 hours/day) and long sleep (≥ 9.0 hours/day) (17). Given the inconsistent results reported in the current literature, the linear dose-response relationship observed in our present study needs to be verified in further prospective cohort studies with a large sample size.

Apart from sleep duration, sleep timing, including wake-up time and bedtime, may also be an important contributor to childhood obesity (11, 37–40). In a recent observational cross-sectional study on 2200 Australian children aged 9–16 years, it was shown that independent of sleep duration, later bedtime/wake-up time was associated with 1.47 times higher risk of obesity compared with the early bedtime/wake-up time pattern (41). In a prospective study of US children, bedtime was found to have a greater impact on childhood obesity than do wake-up time (24). Consistent with previous observations, the present study found that later bedtime (after 10:00 pm) on weekends was associated with abdominal obesity in all children after adjusting for other confounders. Behavioral factors may partly be involved in this association. A late bedtime was associated with less physical activity, more sedentary activities, and higher screen time (41). Besides, a late bedtime was related to increased fat intake and increased intake of energy-dense, nutrient-poor foods as well as sweet drinks (40). Notably, we found that the association between bedtime and abdominal obesity was only observed on weekends. Weekday sleep timing is largely influenced by the school schedule, while weekend sleep timing is fairly aligned with individual preferences. The casual relationship between sleep duration, wake-up time, bedtime, and abdominal obesity needs to be explored in longitudinal studies in the future.

Another interesting finding worthy of note was that after subgroup analyses by age and sex, the close associations between weekend sleep duration, weekend bedtime, and abdominal obesity were stronger among boys and younger age group (7–12 years). Some previous studies also found sex and age differential associations between sleep and overweight/obesity (15, 17, 21, 42). For instance, Duan et al. studied 1008 Chinese children and adolescents found short sleep duration was associated with abdominal obesity in younger girls (6–12 years, OR=2.34, 95% CI: 1.07–5.13) and older boys (13–17 years, OR=2.30, 95% CI: 1.10–4.82), but not in younger boys and older girls (20). In another cross-sectional study of 723 adolescents from the USA, the relationship between sleep duration and BMI was moderated both by sex and grade level, which was evident in middle-school boys (β=−0.32) and girls (β=−0.18) but largely absent in high-school students (21). Sun et al. studied 5773 Japanese children aged 12–13 years indicated that boys may have a higher risk of overweight/obesity when they had the same level of sleep duration as girls (22). The mechanisms to explain these sex and age differences are unclear.

Several limitations should be considered when interpreting our present findings. First, this was a cross-sectional study. Although a close relationship between weekend sleep duration and childhood abdominal obesity has been observed in our study, no cause-effect relationship could be verified due to the study design. Second, sleep duration, wake-up time, and bedtime were assessed by questionnaires, rather than objective assessments, which may result in the risk of bias. However, parent-reported sleep patterns have been shown to have a satisfactory agreement with objective tools in measuring sleep behaviors (43). Third, in our present study, wake-up time and bedtime were obtained to calculate the sleep duration, while the sleep time was not recorded. Although there may have dissociation between bedtime and sleep time especially in older children, no data that might infer directional associations could be found in our study. Fourth, daytime napping and sleepiness, the difference between weekdays and weekends in physical activity were not covered by our questionnaire. Finally, though our present study included a large sample with a wide range of ages, the results might not be representative of the national population.

In conclusion, the present study revealed that sleep duration and bedtime on weekends were different from those on weekdays and were independently associated with the risk of childhood abdominal obesity. Furthermore, decreasing weekend sleep duration was associated with an increased occurrence of abdominal obesity, particularly in boys and children aged 7–12 years. Our findings may have important implications for developing public health policies and effective intervention programs to prevent childhood abdominal obesity. Further longitudinal follow-up and intervention studies are required to investigate the directional of sleep and abdominal obesity among children.

## Data Availability Statement

The original contributions presented in the study are included in the article/**Supplementary Material**. Further inquiries can be directed to the corresponding author.

## Ethics Statement

All subjects were informed about the aims and procedures of the study and written informed consent was received from all children’s parents before they participating in the study. The studies involving human participants were reviewed and approved by the ethics committee of Beijing Children’s Hospital, Capital Medical University. Written informed consent to participate in this study was provided by the participants’ legal guardian/next of kin.

## Author Contributions

All authors helped to perform the research. MJL wrote the manuscript. BC contributed to the project management. QL participated in the interpretation of data. QW, ML, XL, DW, WL, CS, and JC took part in the collection of clinical samples. CG conceived and designed the project as well as revised the manuscript. All listed authors revised the paper critically and approved the final version of the submitted manuscript.

## Funding

This study was funded by the National Key Research and Development Program of China (2016YFC0901505, 2016YFC1305304), The Pediatric Medical Coordinated Development Center of Beijing Hospitals Authority (XTYB201808), and the Beijing Municiple Administration of Hospital Clinical Medicine Development of Special Funding Support (No. ZYLX201821).

## Conflict of Interest

The authors declare that the research was conducted in the absence of any commercial or financial relationships that could be construed as a potential conflict of interest.

## Publisher’s Note

All claims expressed in this article are solely those of the authors and do not necessarily represent those of their affiliated organizations, or those of the publisher, the editors and the reviewers. Any product that may be evaluated in this article, or claim that may be made by its manufacturer, is not guaranteed or endorsed by the publisher.
