# Supplementary material for: Associations Between Sleep Duration, Wake-Up Time, Bedtime, and Abdominal Obesity: Results From 9559 Chinese Children Aged 7–18 Years
Source: Front Endocrinol (Lausanne). 2021 Oct 14;12:735952. doi: 10.3389/fendo.2021.735952 (PMC8552807; doi:10.3389/fendo.2021.735952)
Supplement: Supplementary file 1 [file Table_1.docx]

**Supplementary Table 1 Unconditional logistic regression analysis of associations between weekend sleep duration and abdominal obesity.**

|  | **weekend sleep duration, hours** | | | |
| --- | --- | --- | --- | --- |
| **Measurement** | **<8 hours/day**  **OR (95% CI)** | **8–9 hours/day**  **OR (95% CI)** | **9–10 hours/day**  **OR (95% CI)** | **>10 hours/day**  **OR (95% CI)** |
| Overall |  |  |  |  |
| Model 1 | 2.00 (1.67–2.40) | 1.61 (1.38–1.88) | 1.22 (1.04–1.43) | 1.00 (reference) |
| Model 2 | 2.03 (1.68–2.44) | 1.69 (1.44–1.98) | 1.28 (1.09–1.51) | 1.00 (reference) |
| Boys |  |  |  |  |
| Model 1 | 2.20 (1.71–2.83) | 1.76 (1.42–2.20) | 1.22 (0.97–1.53) | 1.00 (reference) |
| Model 2 | 2.26 (1.74–2.93) | 1.87 (1.49–2.35) | 1.30 (1.03–1.64) | 1.00 (reference) |
| Girls |  |  |  |  |
| Model 1 | 1.81 (1.39–2.36) | 1.45 (1.17–1.81) | 1.23 (0.988–1.54) | 1.00 (reference) |
| Model 2 | 1.70 (1.30–2.23) | 1.50 (1.20–1.81) | 1.28 (1.02–1.88) | 1.00 (reference) |
| 7–12 years old |  |  |  |  |
| Model 1 | 2.41 (1.94–3.01) | 1.65 (1.38–1.97) | 1.25 (1.04–1.49) | 1.00 (reference) |
| Model 2 | 2.37 (1.89–2.98) | 1.73 (1.44–2.08) | 1.31 (1.09–1.58) | 1.00 (reference) |
| 13–18 years old |  |  |  |  |
| Model 1 | 1.47 (1.05–2.06) | 1.50 (1.09–2.07) | 1.15 (0.82–1.61) | 1.00 (reference) |
| Model 2 | 1.54 (1.09–2.17) | 1.55 (1.11–2.16) | 1.20 (0.85–1.69) | 1.00 (reference) |

Multivariate ORs and 95% CIs from unconditional logistic regression models were used
in the analysis.

Model 1: basic model, adjusted for age and gender.
Model 2: further adjusted for parental obesity, parental education, gestational diabetes mellitus, infant breastfeeding, birth weight, siblings, household income based on model 1.
